# Supplementary material for: Dirac fermions at high-index surfaces of bismuth chalcogenide topological insulator nanostructures
Source: Sci Rep. 2016 Feb 5;6:20220. doi: 10.1038/srep20220 (PMC4742872; doi:10.1038/srep20220)
Supplement: Supplementary Information [file srep20220-s1.pdf]

Supplementary Information for

Dirac fermions at high-index surfaces of bismuth chalcogenide topological insulator nanostructures

Naunidh Virk[1] and Oleg V. Yazyev[1,*]

[1]*Institute of Theoretical Physics, Ecole Polytechnique Fédérale de Lausanne (EPFL), CH-1015 Lausanne, Switzerland*

[*] Correspondence and requests for materials should be addressed to O.V.Y. (email: oleg.yazyev@epfl.ch).

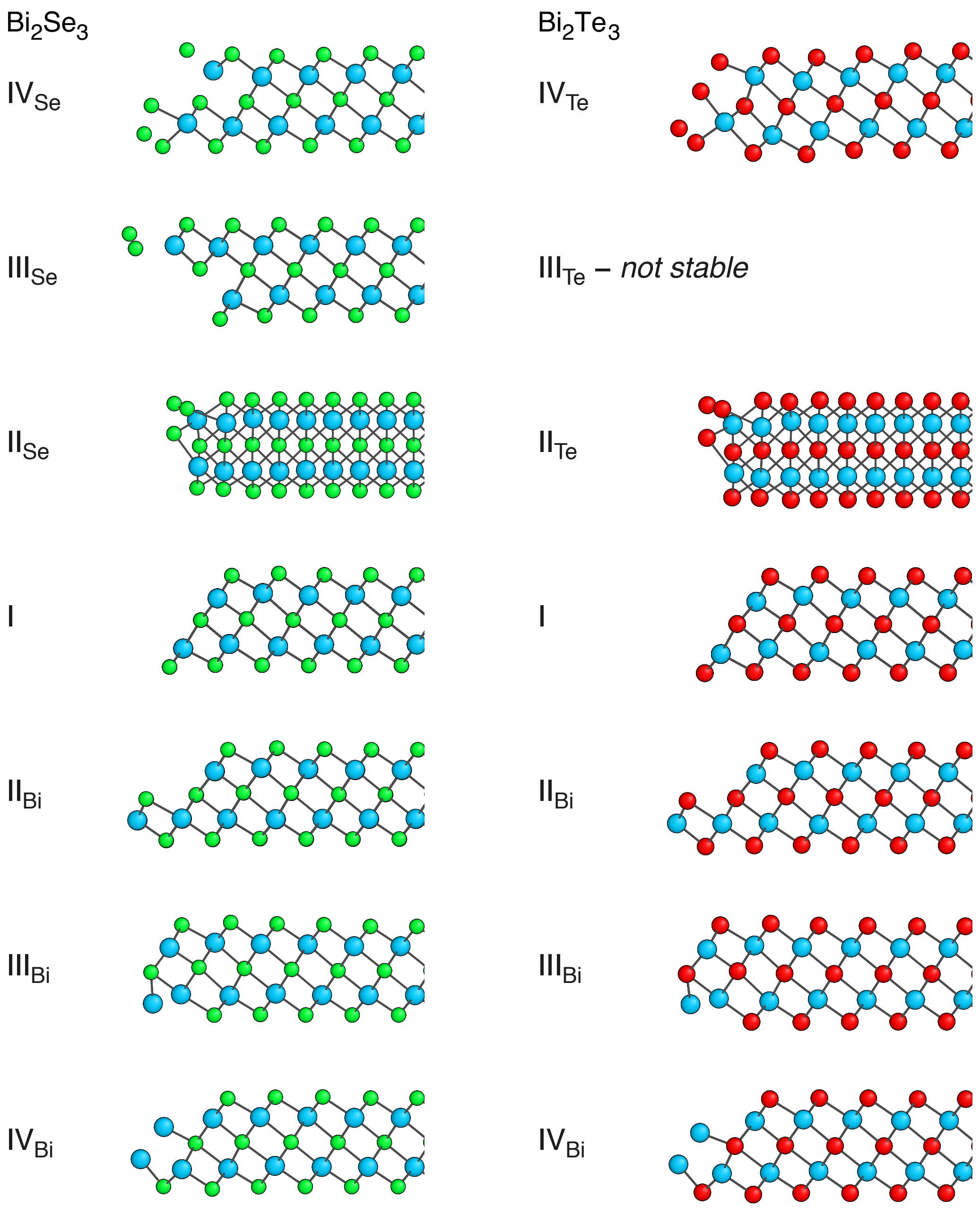

Figure S1 | Atomic structures of QL terminations of bismuth chalcogenide topological insulators. Relaxed atomic structures of stoichiometric and non-stoichiometric QL terminations showing regions of stability (cf. Fig. 2 of the main text).

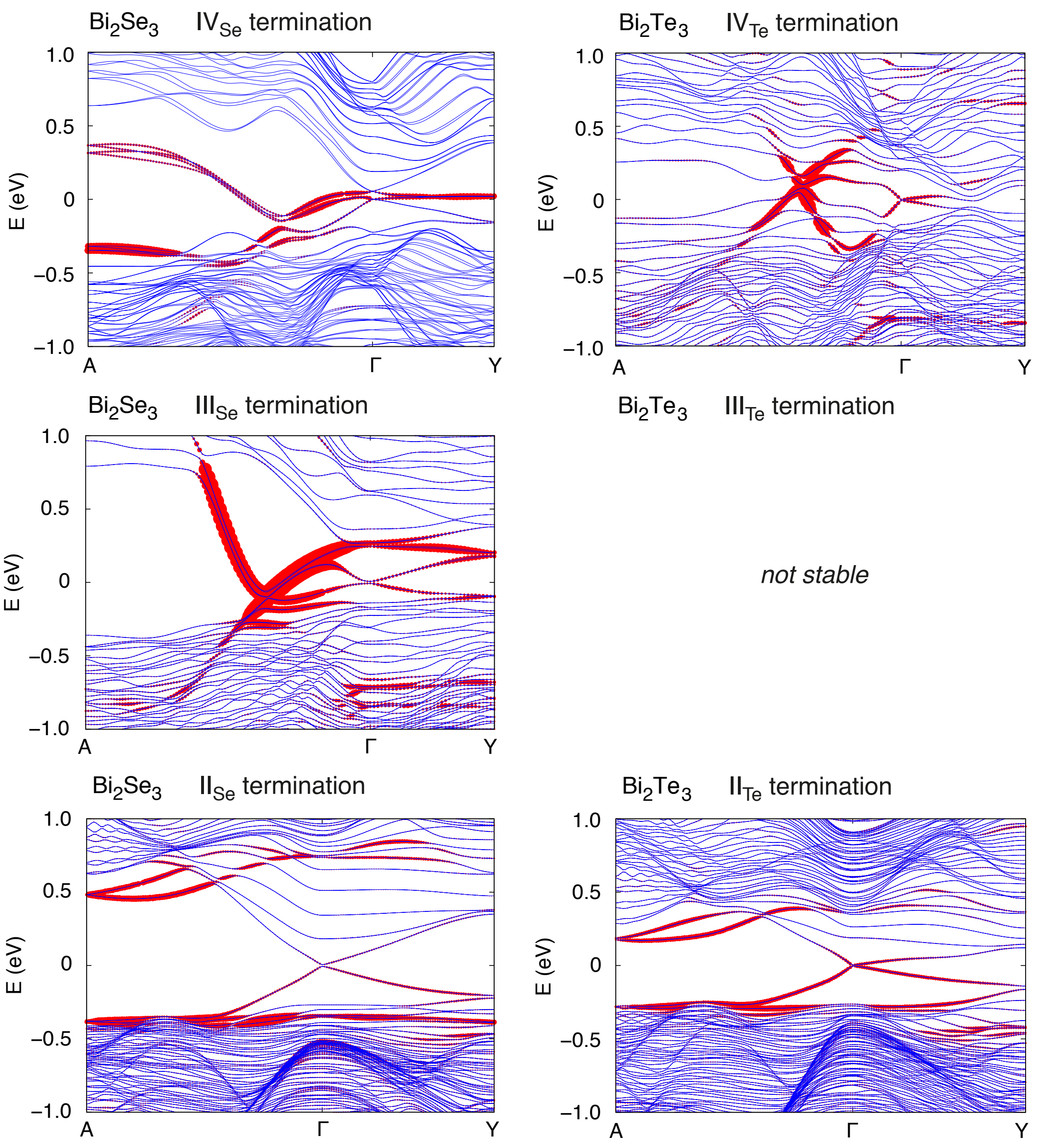

Figure S2 | Band structures of Se- and Te-rich high-index slab models. First-principles band structures of slab models of Se-rich (left) and Te-rich (right) high-index surfaces of Bi_2Se_3 and Bi_2Te_3, respectively, at $\theta = 57.7°$. Points A and Y correspond to the Brillouin zone boundaries along directions defined by reciprocal lattice vectors associated with the real-space unit vectors of the surface. The size of red symbols reflects the magnitude of the inverse participation ratio (IPR).

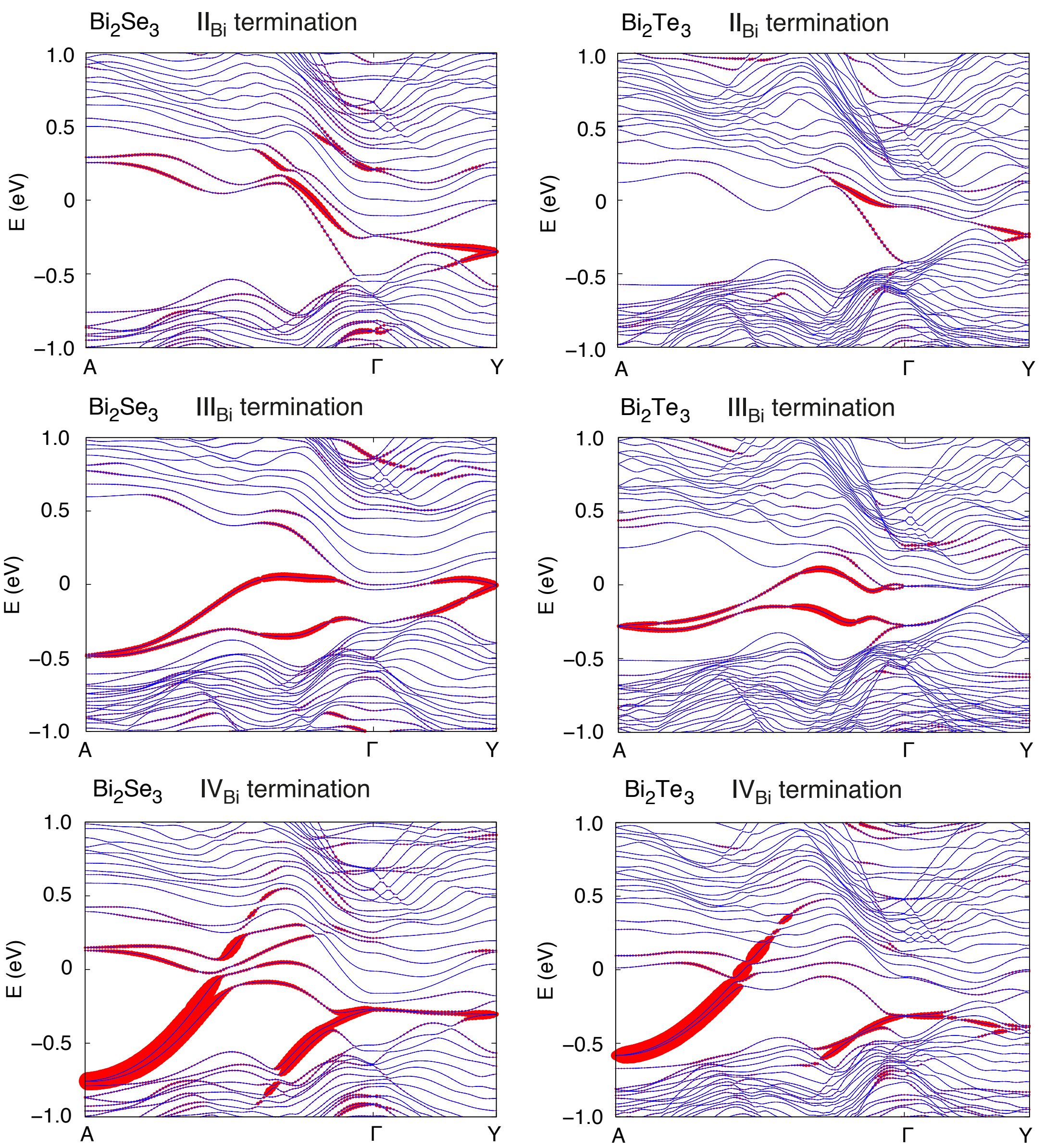

Figure S3 | Band structures of Bi-rich high-index slab models. First-principles band structures of slab models of Bi-rich high-index surfaces of Bi_2Se_3 and Bi_2Te_3 at θ = 57.7°. Points A and Y correspond to the Brillouin zone boundaries along directions defined by reciprocal lattice vectors associated with the real-space unit vectors of the surface. The size of red symbols reflects the magnitude of the inverse participation ratio (IPR).
